# Aqueous column changes in the episcleral veins after the instillation of ripasudil versus latanoprost: a randomized, double-blind, crossover clinical trial

**DOI:** 10.1038/s41598-022-19271-9

**Published:** 2022-09-10

**Authors:** Marie Suzuki, Yohei Suzuki, Ryohei Komori, Yusuke Orii, Shogo Arimura, Kentaro Iwasaki, Yoshihiro Takamura, Masaru Inatani

**Affiliations:** Department of Ophthalmology, Faculty of Medical Sciences, University of Fukui, 23-3 Shimoaizuki, Matsuoka, Eiheiji, Yoshida, Fukui 910-1193 Japan

**Keywords:** Randomized controlled trials, Blood flow, Glaucoma

## Abstract

To investigate whether the topical administration of ripasudil ophthalmic solution enhances aqueous outflow in the episcleral vein of the human eye. Two-sequence, prospective, randomized, double-blind, crossover trial. Sixteen eyes of 16 healthy participants were recruited in this study. Participants were randomized into one of the two crossover sequences to the instillation of ripasudil or the control drug, latanoprost, followed by a washout period of more than 2 days, and crossed over to the alternative instillation. The aqueous columns in the episcleral veins were recorded using a video capture system connected to a slit-light microscope (hemoglobin video imaging) before and 2 and 8 h after the instillation. Comparisons between ripasudil and latanoprost for the changes of the aqueous column width after the instillation. Two hours after the instillation, the ripasudil group had significantly greater dilation of the aqueous column width than the latanoprost group. Eight hours after the instillation, the ripasudil group had significantly greater dilation of the aqueous column width than the latanoprost group. Hemoglobin video imaging revealed that the topical administration of ripasudil ophthalmic solution enhanced aqueous outflow in the episcleral vein of the human eye.

## Introduction

Lowering intraocular pressure (IOP) is the only evidence-based treatment for glaucoma^1^. The initial treatment for lowering IOP in the eyes with open-angle glaucoma is medical therapy using ophthalmic solution, rather than surgical therapy. Glaucoma ophthalmic solution is designed to reduce IOP by either decreasing the production of aqueous humor or promoting its drainage. Among the various types of glaucoma ophthalmic solutions, Rho-kinase (ROCK) inhibitors improve the drainage of the aqueous humor through the trabecular conventional aqueous outflow pathway. The conventional outflow pathway is the main outflow pathway because it drains 80–90% of the volume of aqueous humor. The pathway consists of the trabecular meshwork, Schlemm’s canal, collector channel, and episcleral veins. ROCK inhibitors, such as ripasudil and netarsudil, are believed to reduce the resistance of the conventional outflow pathway by altering the morphology of the trabecular meshwork and dilating Schlemm’s canal and the episcleral veins^2,3^.

In vitro experiments, animal studies, and histological observations using ROCK inhibitors have indicated that ROCK inhibitors induce structural and functional changes in the conventional outflow pathway^4,5^. However, few studies have shown a change in the conventional outflow pathway in human eyes caused by the ROCK inhibitor ophthalmic solution under physiological conditions. Recently, optical coherence tomography angiography (OCTA) in the anterior segment has been used to visualize red blood cell components in the superficial and deep vessels of the conjunctiva and sclera. The deep vessels correspond to those in the sclera, including the episcleral vein, which is the downstream of the collector channel in the main pathway. Interestingly, ripasudil ophthalmic solution increases the vessel density in the sclera^6^. However, OCTA detects the flow of red blood cells in the vessel in the sclera, not the aqueous humor in the episcleral vein, and cannot show the direction of the flow in the episcleral vein. Therefore, it is difficult to evaluate whether ROCK inhibitors enhance the volume of the aqueous humor in the episcleral vein.

Another approach to measure the aqueous outflow in the conventional outflow pathway is live imaging of the outflow of the aqueous humor in the episcleral vein through slit-lamp microscopy. The aqueous outflow was first visualized in the 1940s as a clear central laminar flow in the episcleral vein^7^. A recent digital video imaging system connected to slit-lamp microscopy has quantified the aqueous outflow in the human eye. The live imaging, which is called hemoglobin video imaging, has revealed that surgical therapies, including selective laser trabeculoplasty and iStent Inject®, enhance the aqueous outflow in the episcleral vein^8,9^. Because these surgical therapies reduce IOP, improving the outflow resistance in the conventional outflow pathway, it is possible that the live imaging system can visualize the aqueous outflow change in the episcleral vein of the human eye after the administration of the ROCK inhibitor.

This study aimed to confirm the enhanced aqueous humor outflow in the episcleral vein in human eyes after the instillation of a ROCK inhibitor, ripasudil, using hemoglobin video imaging. To identify whether the change provided by the ROCK inhibitor was unique, we conducted a crossover clinical trial comparing it with latanoprost ophthalmic solution that reduces IOP through the enhanced uveoscleral aqueous outflow.

## Methods

### Participant selection

This study was conducted in accordance with the tenets of the Declaration of Helsinki. This study was approved by the Institutional Review Board of the Fukui University Hospital, Fukui, Japan. This study was registered with the University Hospital Medical Information Network Clinical Trials Registry of Japan (identifier: University Hospital Medical Information Network 000046665, date of access and registration: January 18, 2022). Participants were recruited between January 18, 2022, and March 14, 2022. Data were collected from 16 eyes of 16 healthy adults. Written consent was obtained from all participants after an adequate informed consent. Volunteers who were treated with glaucoma medications, with allergic conjunctivitis, or with other ocular diseases were excluded from the study. Volunteers who were pregnant or lactating or had the possibility of pregnancy during the study period were also excluded. Contact lens users kept their lenses off during the study.

### Crossover drug administration

**Figure 1:**
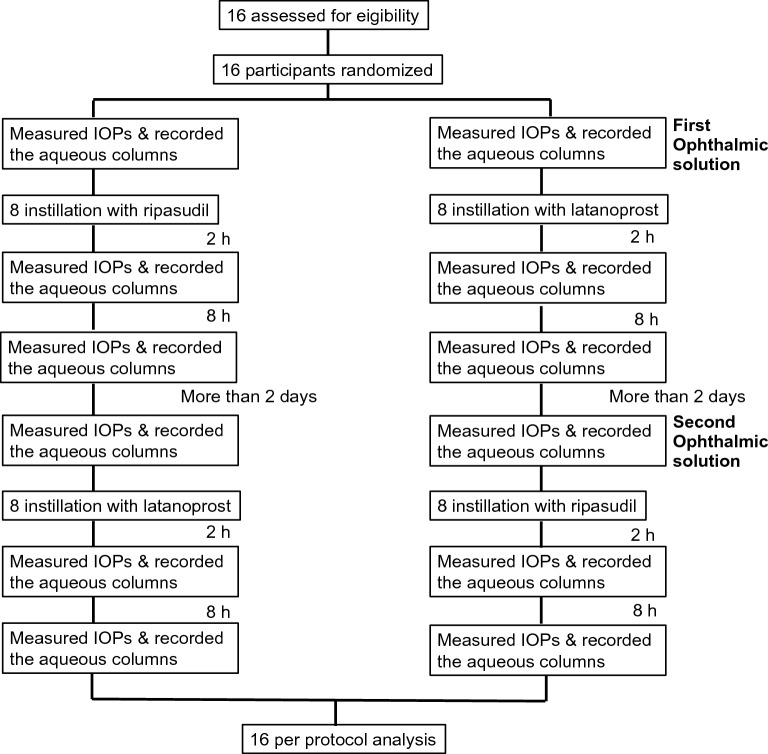
Flowchart of participant progress in this study. IOP, intraocular pressure.

A 0.4% ripasudil hydrochloride hydrate ophthalmic solution (GLANATEC^®^, Kowa, Nagoya, Japan) and a 0.005% latanoprost ophthalmic solution (Xalatan^®^, Viatris, Tokyo, Japan) were used in this study. Healthy volunteers were randomized at a 1:1 ratio to one of the two crossover sequences to the instillation with ripasudil or latanoprost ophthalmic solution. After a washout period of more than 2 days, they were crossed over to the alternative instillation (Fig. 1).

This study was conducted in a double-blind manner. A random number table was used to determine the ophthalmic solution that was first instilled to each volunteer. The investigator, who randomly assigned volunteers, instilled ophthalmic solutions without letting them know which drugs were instilled. Another investigator measured IOPs with applanation tonometry and recorded the aqueous columns in the episcleral veins with a video capture system connected to the slit-light microscope (hemoglobin video imaging) before and 2 and 8 h after the instillation.

### Measurement of aqueous column

The width of the aqueous column was determined according to a previous study^8^. Briefly, we set a 45× magnification of the slit-lamp microscope. A green filter (between 505 and 575 nm wavelengths) was used to visualize episcleral veins and aqueous columns. We used an iPhone 11 (Apple, Cupertino, CA) to record the video movies. The iPhone 11 was connected to the eyepiece part of the slit lamp and set to 1.5× magnification. The images were captured in 4K at 60 frames per second. When we analyzed the captured images, we alternately skipped one frame out for the 60 frames and then analyzed 30 frames per second. Images were recorded four and nine times to record continuous aqueous flow in the episcleral vein. We selected the images obtained immediately after blinking because the aqueous flow tended to slow down without blinking. The captured images were converted to the Audio Video Interleave format, and the files were applied to ImageJ (available at http://imagej.nih.gov/ij/). The width of the aqueous column was analyzed using the ImageJ software. We used the StackReg plugin to compensate for camera shake in video. The width of the aqueous column in the episcleral vein was defined as the distance between the minimum intensity (Supplemental Fig. 1) because of the cross-sectional view of the vein. The diameter was measured in 30-frame slices by two independent observers (MS and YS), and the average value was calculated.

The flow rate was also calculated using the following formula described in the previous report^8^:

$$Rij(n) = E[(pij (t){\text{-}}{\upmu })(Pij( t + n){\text{-}}{\upmu })]/ \sigma ^{\land}{2}$$

where Rij(n) denotes the autocorrelation function value for a pixel at position (i, j) at a frame delay value t, µ is the mean pixel value in the segmented image, and σ is the standard deviation of the pixel values in the segment.

### Data collection

The right eye was selected as the priority in the present study. Aqueous columns in the episcleral veins were frequently observed in the inferonasal quadrant^10^. If no aqueous columns were observed, images of the aqueous columns were recorded in the inferotemporal quadrant. If no aqueous columns were present in the inferior quadrant, the left eye was selected. Participants’ data included age, sex, laterality of the eye, IOP, and vein location.

### Primary outcome measures

The primary outcome measures were the comparisons between ripasudil and latanoprost for the changes in the aqueous column width after the instillation.

### Secondary outcome measures

The secondary outcome measures were as follows; the association between the width of the aqueous column and the rate of IOP reduction from the baseline IOP; the changes of the flow rate after the drug administration; the occurrence of the adverse events during the study.

### Statistical analyses

Data are presented as mean ± standard deviation. A comparison of the change in the aqueous column width between the two types of ophthalmic solutions at 2 and 8 h after the instillation was performed using a linear mixed model. The sequences, ophthalmic solutions, and periods were included in the model as fixed effects. The participants within a sequence were included in the model as random effects. Comparison of the change between baseline and 2 or 8 h after instillation was performed using the Wilcoxon signed-rank test. Statistical significance was set at p < 0.05.

## Results

### Participant characteristics

All 16 healthy volunteers completed this study. The characteristics of the participants and their IOPs are summarized in Table 1.

**Table 1:** Baseline characteristics.

	Ripasudil to latanoprost group, 8 participants	Latanoprost to ripasudil group, 8 participants	p value
Male, eyes (%)	2 (25%)	3 (37.5%)	p = 0.590*
Age, years	26 ± 5.1	26 ± 4.2	p = 1^†^
Right, eyes (%)	8 (100%)	6 (75%)	p = 0.131*
Baseline IOP, mm Hg	14.0 ± 2.1	12.5 ± 1.4	p = 0.065^‡^
The aqueous veins in inferonasal, eyes (%)	7 (87.5%)	8 (100%)	p = 0.302*

Age and IOP; intraocular pressure are mean ± SD.

*χ^2^ test.

^†^Unpaired t-test.

^‡^Wilcoxon rank sum test.

### Primary outcome measures

**Figure 2:**
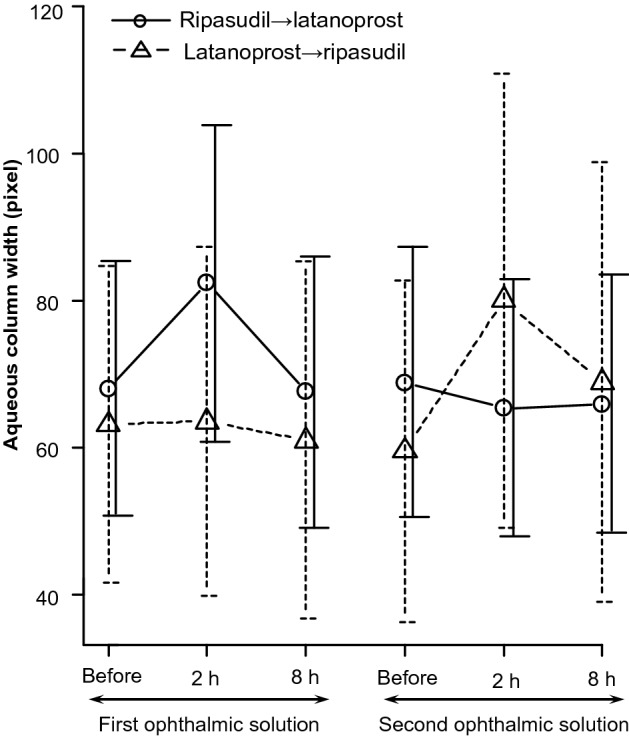
Mean change in aqueous column width before and after the instillation of each group.

Figure 2 shows the histograms of the aqueous column width of the ripasudil to latanoprost and latanoprost to ripasudil groups during the study.

The aqueous column widths 2 h after the instillation of the first ophthalmic solution in the ripasudil to latanoprost group (p = 0.012, Wilcoxon signed-rank test) and at 2 h after the instillation of the second ophthalmic solution in the latanoprost to ripasudil group (p = 0.012, Wilcoxon signed-rank test) were significantly larger than the width before the instillation.

Table 2 shows the changes in the aqueous column width after the instillation of each ophthalmic solution.

**Table 2:** Comparison of mean width of aqueous column.

	Before, baseline	2 h, ripasudil	8 h, ripasudil	Before, baseline	2 h, latanoprost	8 h, latanoprost
Width of the aqueous columns, pixel	63.7 ± 19.6	81.2 ± 24.9	68.2 ± 23.2	66 ± 18.9	64.5 ± 19.4	63.4 ± 19.9
Percent change in width from baseline (%)		28.8 ± 16.3	7.6 ± 12.6		−2.5 ± 5.7	−4.3 ± 8.1
p value*		p < 0.001*	p = 0.034*		p = 0.187	p = 0.056
Percent change in width between ripasudil and latanoprost p value^‡^					p < 0.001^‡^	p = 0.0212^‡^

*Within-group comparison to baseline.

^‡^Between group comparison.

**Figure 3:**
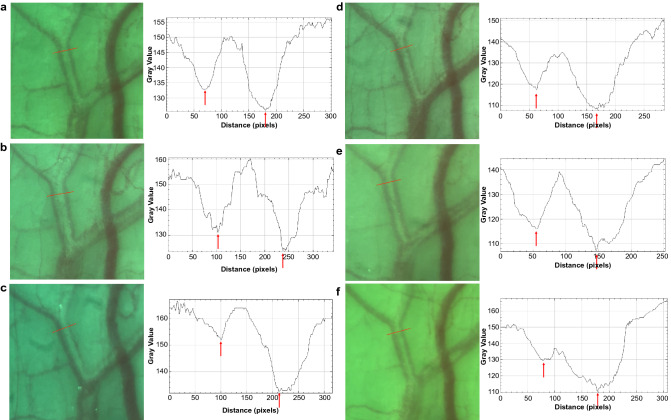
The graph of a representative case with ripasudil and latanoprost instillations. (**a**) before ripasudil instillation. (**b**) 2 h after ripasudil instillation. (**c**) 8 h after ripasudil instillation. (**d**) before latanoprost instillation. (**e**) 2 h after latanoprost instillation. (**f**) 8 h after latanoprost instillation. The line presents the measurement position. The arrow presents the minimum intensity.

The aqueous column width was significantly larger 2 (p < 0.001, Wilcoxon signed-rank test) and 8 h (p = 0.034, Wilcoxon signed-rank test) after the instillation of ripasudil than that at baseline. In contrast, no significant differences in aqueous column width were observed after the instillation of latanoprost. Representative images and graphs reflecting the signal intensity of the episcleral veins are shown in Fig. 3. Two hours after the instillation of ripasudil, the diameter of the aqueous column became wider than that of the baseline and was restored 8 h after the instillation (Fig. 3a–c). However, the diameter of the aqueous column appeared to remain unchanged after the instillation of latanoprost (Fig. 3d–f).

Furthermore, 2 h after the instillation of ophthalmic solutions, the percent change in the aqueous column width was significantly larger after the instillation of ripasudil than after the instillation of latanoprost (p < 0.001, liner mixed model). Eight hours after the instillation of ophthalmic solutions, the percent change of the aqueous column width was also significantly larger after the instillation of ripasudil than after the instillation of latanoprost (p = 0.0212, linear mixed model).

### Intraocular pressure in secondary outcome measures

IOPs significantly decreased 2 (p < 0.001, Wilcoxon signed-rank test) and 8 h (p = 0.013, Wilcoxon signed-rank test) after the instillation of ripasudil (Table 3).

**Table 3:** Intraocular pressures before and after the administration of ripasudil or latanoprost.

	Baseline	2 h	8 h
Ripasudil (mm Hg)	12.8 ± 2.5	10.6 ± 2.1	11.4 ± 2.2
p value*		p < 0.001*	p = 0.013*
Latanoprost (mm Hg)	12.6 ± 1.7	12.3 ± 2.2	10.3 ± 2.0
p value*		p = 0.533	p < 0.001*

Intraocular pressure is mean ± standard deviation.

*Within-group comparison to baseline.

IOPs were also significantly decreased 8 h (p < 0.001, Wilcoxon signed-rank test) after the instillation of latanoprost, whereas the difference in IOPs between baseline and 2 h after the instillation of latanoprost was not significant (p = 0.533, Wilcoxon signed-rank test).

The association between IOP changes and changes in the aqueous column width 2 h after the instillation of ripasudil is shown in Supplemental Fig. 2.

No significant correlation was found between these two parameters (p = 0.537).

### Flow rates in secondary outcome measures

Flow rates at baseline and 2 and 8 h after the instillation of the two drugs were plotted for each participant. The graph shows the speed of red blood cell flow (Supplemental Fig. 3).

No consistent trends were observed at any time point when the two drugs were changed in the type of ophthalmic solution or time points.

### Adverse events in secondary outcome measures

There were no adverse events, including blepharitis, allergic conjunctivitis, keratitis, iritis, visual disturbance, or anaphylactic shock during the study.

## Discussion

This study aimed to investigate whether the topical instillation of ripasudil ophthalmic solution enhances aqueous outflow in the episcleral vein of the human eye. The hemoglobin video imaging demonstrated that the dilation of the aqueous column width after the instillation of ripasudil was significantly larger than the dilation of the aqueous column width after the instillation of latanoprost (p < 0.001 at 2 h and p = 0.0212 at 8 h). During the study, the aqueous column width significantly increased after the instillation of ripasudil (p < 0.001 at 2 h and p = 0.034 at 8 h), whereas the aqueous column width did not increase after the instillation of latanoprost. The data proved our hypothesis that ripasudil ophthalmic solution enhances aqueous outflow in the episcleral vein of the human eye, clearly indicating a difference in IOP-lowering mechanisms between ripasudil and latanoprost.

Previous studies have reported structural and functional changes in the episcleral vein after the instillation of ROCK inhibitors in ex vivo and animal experiments. Ren et al. reported increased aqueous humor outflow in the episcleral veins of enucleated human eye perfused with a ROCK inhibitor. Kiel et al. invasively evaluated the decreased pressure and dilation of the aqueous veins after the instillation of a ROCK inhibitor in rabbit eyes. Although various reports using in vitro^11,12^, ex vivo^5^, and animal models^3,4^ have shown the effect of ROCK inhibitors on aqueous outflow through the conventional outflow pathway, little data supporting this effect have been obtained in humans. Recently, OCTA of the anterior segment has shown supportive data among healthy volunteers. The vessel density in the sclera is enhanced by the instillation of ripasudil ophthalmic solution^6^. OCTA detects the movement of red blood cells in the vessels. The increased vessel density after ripasudil administration in their study reflects the increased flow of red blood cells in the episcleral veins. If the vessels contained the aqueous column, the increased vessel density would be associated with the increased flow of aqueous humor. However, OCTA has some limitations in the measurement of enhanced aqueous outflow. First, OCTA does not detect the direction of red cell blood flow in the episcleral vein. It means that reversed flow of the red blood cells in the episcleral vein is also detected. Second, OCTA does not distinguish arteries from veins. The vessels detected by OCTA include arteries in the deep sclera. Third, OCTA also includes the signal of the episcleral veins that do not contain aqueous humor. Forth, the increased vessel density is related to the vessel dilation caused by ripasudil. Therefore, because our present study directly quantified the aqueous column width using hemoglobin video imaging, our study confirmed the enhanced aqueous outflow in the episcleral vein after the administration of ROCK inhibitor.

The topical administration of prostaglandin analogs causes conjunctival hyperemia^13^. Regarding the mechanism for conjunctival hyperemia, animal studies have suggested that prostaglandin promotes vascular endothelial cells to release nitric oxide. Vessel dilation caused by nitric oxide is associated with conjunctival hyperemia^14^. Topically instilled ROCK inhibitors also cause conjunctival hyperemia^15,16^. Because ROCK inhibitors induce smooth muscle relaxation in the vessels, hyperemia is caused by vessel dilation. Although the two drugs cause vessel dilation, the increased aqueous column width in the episcleral vein is unique in ripasudil instillation, indicating the effect of increased aqueous outflow through the conventional outflow pathway.

Compared to the baseline, the aqueous column width was higher 2 h after the instillation of ripasudil and was restored 8 h after the instillation, although it was still significantly larger at 8 h than at baseline. Peak IOP reduction was observed 2 h after the instillation of ripasudil^17^. The time-dependent change in the aqueous column width was consistent with the duration of drug efficiency. Netarsudil ophthalmic solution, another ROCK inhibitor, has a higher efficiency than ripasudil ophthalmic solution^18,19^. Because of once-daily instillation, the time-dependent change in the aqueous column width might be different from that of ripasudil.

Although selective laser trabeculoplasty increased the flow rate and aqueous column width in the episcleral vein^8^, ripasudil did not increase the flow rate in the present study. The discrepancy between the two interventions might be due to differences in IOP-lowering mechanisms. Selective laser trabeculoplasty enhances aqueous humor outflow from the trabecular meshwork^20^. The expanded volume of aqueous humor flowing into the episcleral vein might increase the aqueous column width and aqueous flow rate. ROCK inhibitors not only enhance aqueous humor outflow in the trabecular meshwork but also reduce outflow resistance, relaxing smooth muscle in the episcleral vein^5,21^. The dilation of episcleral veins might attenuate the flow rate in the episcleral vein, although the aqueous volume was increased by ripasudil. Another explanation may be the differences in the recruited cases. Individuals who were treated with selective laser trabeculoplasty in a previous study had high IOPs^8^. By contrast, our present study recruited healthy volunteers with a mean IOP before the instillation of 12.8 mm Hg. The relatively low IOPs in these individuals might result in an unchanged aqueous flow rate after ripasudil instillation. Regarding low IOPs in the recruited participants, we also found no significant correlation between the change in aqueous column width and IOP reduction after ripasudil instillation. The IOP reduction provided by glaucoma medication is less in eyes with normal IOPs than in eyes with high IOPs^22^. The small IOP changes might result in no significant correlation between these two parameters. Another explanation might be related to quantification for the aqueous column width in one episcleral vein of each volunteer. IOP reduction caused by ripasudil is thought to be determined by the total of the enhanced aqueous outflow in all the episcleral veins after the administration. No significant correlation between these two parameters might have occurred because our present study examined the change of the aqueous column width in one episcleral vein of each volunteer.

The present study has some limitations. First, the change in the aqueous column width in the episcleral vein was measured up to 8 h after the instillation. We could not measure the change in the aqueous column width more than 8 h after the instillation because of the schedules of healthy volunteers. The aqueous column width 8 h after the instillation of ripasudil was still significantly higher than that at baseline, indicating that the effect lasted for 8 h. Our study could not determine how long ripasudil is effective for enhancing the aqueous column width. Second, this procedure cannot quantify the flow volume of the aqueous humor in the episcleral vein, although the change in the aqueous column width reflects the change in the flow volume^8^. Hence, another methodology is required for the quantification. Third, in this study, we did not confirm the changes in the aqueous column width after the ripasudil administration in glaucomatous eyes. The function of the trabecular meshwork and collector channels is impaired in glaucomatous eyes. Ripasudil administration might less enhance the dilation of the aqueous humor column in glaucoma eyes than in healthy eyes. A further additional study would be required to examine whether ripasudil could also improve the aqueous column width of patients with various glaucoma-subtypes as shown in the present study.

## Conclusion

In conclusion, hemoglobin video imaging revealed the dilation of the aqueous column width in the episcleral veins of human eyes after the topical instillation of ripasudil. The aqueous column width was significantly higher after the instillation of ripasudil compared to the baseline before and after the instillation of latanoprost. These data indicate that the ROCK inhibitor enhances aqueous outflow through the conventional outflow pathway in the human eye.

## Supplementary Information

Supplementary Figure S1.

Supplementary Figure S2.

Supplementary Figure S3.

## Data Availability

The data that support the findings of this study are available from the corresponding author, [M.S.], upon reasonable request.

## References

[1] Gedde SJ (2021). Primary open-angle glaucoma preferred practice pattern^®^. Ophthalmology.

[2] Kaneko Y (2016). Effects of K-115 (Ripasudil), a novel ROCK inhibitor, on trabecular meshwork and Schlemm’s canal endothelial cells. Sci. Rep..

[3] Li G (2016). Visualization of conventional outflow tissue responses to netarsudil in living mouse eyes. Eur. J. Pharmacol..

[4] Kiel JW, Kopczynski CC (2015). Effect of AR-13324 on episcleral venous pressure in Dutch Belted rabbits. J. Ocul. Pharmacol. Ther..

[5] Ren R (2016). Netarsudil increases outflow facility in human eyes through multiple mechanisms. Invest. Ophthalmol. Vis. Sci..

[6] Akagi T (2020). Short-term effects of different types of anti-glaucoma eyedrop on the sclero-conjunctival vasculature assessed using anterior segment OCTA in normal human eyes: A pilot study. J. Clin. Med..

[7] Ascher KW (1942). The aqueous veins. Am. J. Ophthalmol..

[8] Tasneem Z (2019). Hemoglobin video imaging provides novel in vivo high-resolution imaging and quantification of human aqueous outflow in patients with glaucoma. Ophthalmol. Glaucoma.

[9] Bostan C, Harasymowycz P (2017). Episcleral venous outflow: A potential outcome marker for iStent surgery. J. Glaucoma.

[10] De Vries S (1947). Zichtbare Afvoer van het Kamerwater.

[11] Koga T (2006). Rho-associated protein kinase inhibitor, Y-27632, induces alterations in adhesion, contraction and motility in cultured human trabecular meshwork cells. Exp. Eye Res..

[12] Okamoto M (2020). Rho-associated protein kinase inhibitor induced morphological changes in type VI collagen in the human trabecular meshwork. Br. J. Ophthalmol..

[13] Giuffrè G (1985). The effects of prostaglandin F2 alpha in the human eye. Graefes Arch. Clin. Exp. Ophthalmol..

[14] Astin M, Stjernschantz J (1997). Mediation of prostaglandin F2 alpha-induced ocular surface hyperemia by sensory nerves in rabbits. Curr. Eye Res..

[15] Tanihara H (2008). Intraocular pressure-lowering effects and safety of topical administration of a selective ROCK inhibitor, SNJ-1656, in healthy volunteers. Arch. Ophthalmol..

[16] Tanihara H (2013). Phase 1 clinical trials of a selective Rho kinase inhibitor, K-115. JAMA Ophthalmol..

[17] Isobe T (2014). Effects of K-115, a rho-kinase inhibitor, on aqueous humor dynamics in rabbits. Curr. Eye Res..

[18] Mehran NA (2020). New glaucoma medications: Latanoprostene bunod, netarsudil, and fixed combination netarsudil-latanoprost. Eye (Lond).

[19] Levy B, Ramirez N, Novack GD, Kopczynski C (2015). Ocular hypotensive safety and systemic absorption of AR-13324 ophthalmic solution in normal volunteers. Am. J. Ophthalmol..

[20] Beltran-Agullo L (2013). The effect of selective laser trabeculoplasty on aqueous humor dynamics in patients with ocular hypertension and primary open-angle glaucoma. J. Glaucoma.

[21] McDonnell F, Dismuke WM, Overby DR, Stamer WD (2018). Pharmacological regulation of outflow resistance distal to Schlemm’s canal. Am. J. Physiol. Cell Physiol..

[22] Tanihara H, Kakuda T, Sano T, Kanno T, Gunji R (2020). Safety and efficacy of ripasudil in Japanese patients with glaucoma or ocular hypertension: 12-month interim analysis of ROCK-J, a post-marketing surveillance study. BMC Ophthalmol..

