# Supplementary material for: Aqueous column changes in the episcleral veins after the instillation of ripasudil versus latanoprost: a randomized, double-blind, crossover clinical trial
Source: Sci Rep. 2022 Sep 10;12:15255. doi: 10.1038/s41598-022-19271-9 (PMC9464201; doi:10.1038/s41598-022-19271-9)
Supplement: Supplementary file 1 — Supplementary Figure S1. [file 41598_2022_19271_MOESM1_ESM.pdf]

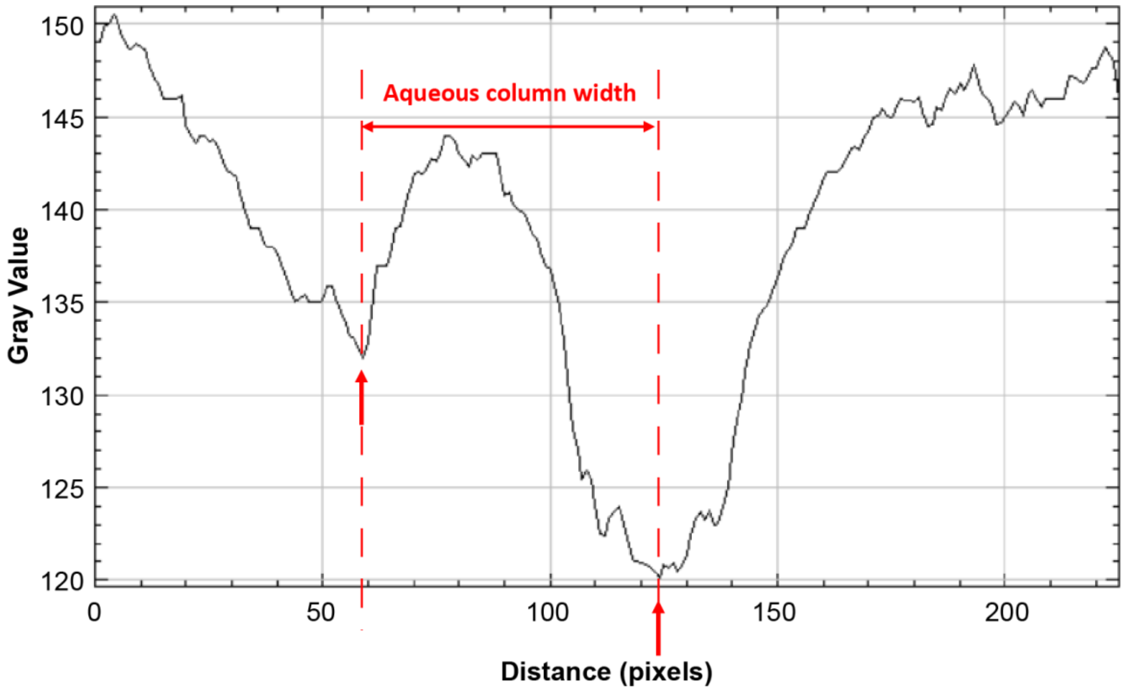

Supplemental Figure 1. The diameter of the aqueous column in the episcleral vein was defined as the distance between the minimum intensity.
